# Bridging the Gap: A Qualitative Study Exploring the Impact of the Involvement of Researchers With Lived Experience on a Multisite Randomised Control Trial in the National Probation Service in England and Wales

**DOI:** 10.1111/hex.70162

**Published:** 2025-02-04

**Authors:** Elizabeth Simes, Stephen Butler, Elizabeth Allison, Barbara Barrett, Anthony Bateman, Angus Cameron, Mike Crawford, Alison Frater, Zoe Hoare, Mary McMurran, Paul Moran, Stephen Pilling, James Wason, Jessica Yakeley, Peter Fonagy

**Affiliations:** ^1^ Research Department of Clinical, Educational and Health Psychology University College London London UK; ^2^ Anna Freud National Centre for Children and Families London UK; ^3^ Psychology Department University of Prince Edward Island Charlottetown Prince Edward Island Canada; ^4^ Institute of Psychiatry, Psychology and Neuroscience, King's College London London UK; ^5^ National Probation Service London Division London UK; ^6^ Centre for Mental Health, Imperial College London UK; ^7^ School of Law, Royal Holloway University of London London UK; ^8^ NWORTH Clinical Trials Unit Bangor University Bangor UK; ^9^ Institute of Mental Health University of Nottingham Nottingham UK; ^10^ Centre for Academic Mental Health, Population Health Sciences Department, Bristol Medical School University of Bristol Bristol UK; ^11^ Population Health Sciences Institute Newcastle University Newcastle upon Tyne UK; ^12^ Portman Clinic, Tavistock and Portman NHS Foundation Trust London UK

**Keywords:** antisocial personality disorder, lived experience, national probation service, patient and public involvement, peer researchers, randomised control trial

## Abstract

**Introduction:**

Methodological and ethical arguments support the involvement of individuals with lived experience in research to reduce engagement barriers and ensure those directly affected by studies contribute to knowledge generation. However, there is limited evidence on the impact of including researchers with lived experience of serving a prison or community sentence in clinical trials. This qualitative study aimed to explore the value of involving researchers with lived experience of the criminal justice system as data collectors in the Mentalization for Offending Adult Males (MOAM), a multisite RCT conducted in the National Probation Service in England and Wales.

**Methods:**

Semi‐structured interviews were conducted with 30 trial participants and 17 key stakeholders, either in person or via telephone. The interviews were transcribed verbatim and analysed thematically.

**Findings:**

Five themes emerged for trial participants and 11 for key stakeholders. For some, lived experience researchers helped overcome engagement barriers by fostering common ground with participants who were serving a prison or community sentence during recruitment. Participants reported that the involvement of lived experience researchers enhanced the study by facilitating knowledge transfer in certain instances. However, their inclusion did not eliminate all barriers and, for some participants, introduced new challenges to engagement.

**Conclusion:**

Forensic lived experience researchers bridged the gap by fostering trust between data collectors and participants. Future studies should ensure that lived experience researchers receive adequate clinical supervision to support their role. The adopted methodology challenged assumptions about knowledge generation and stereotypes associated with being an ex‐offender, benefiting both lived experience and traditional researchers.

**Patient or Public Contribution:**

The study was developed in collaboration with User Voice (charity number: 1136047), who contributed to the study's design and conduct. The service user organisation co‐designed the interview schedule and directed the protocol for participant payments, emphasising a consistent approach to avoid tokenism and ensure equal recognition of all contributions. The dissemination plan was developed in partnership with individuals with lived experience of the criminal justice system.

## 1 Introduction

The value of knowledge gained through experience can be traced to the rise of the Disability Studies movement in the 1960s in Canada and its subsequent establishment in UK healthcare research from the 1990s onwards. This approach has been shaped by government policy, influencing how healthcare research is conducted [1, 2]. Many UK funding bodies now mandate Patient and Public Involvement (PPI) in all grant applications [3]. The National Institute for Health Research defines this involvement as research conducted “with” or “by” members of the public rather than “to,” “about,” or “for” them [4].

Involving individuals with lived experience of the research topic is believed to enhance the quality of research by incorporating diverse perspectives [5]. Moreover, an ethical argument underpins the inclusion of such individuals, as they are directly affected by the research outcomes. The Salzburg Seminar, “Through the Patient's Eyes,” encapsulated this argument with the principle: “nothing about me, without me” [6]. Empowerment through participation is particularly vital for populations that are challenging to engage in healthcare research, such as those involved in the criminal justice system.

The literature highlights how PPI in NHS clinical trials and longitudinal studies can positively influence recruitment rates and participant engagement [7, 8, 9]. In the criminal justice system, the value of service user involvement in aiding rehabilitation is well documented and often conceptualised as the role of the “wounded healer” [10], which supports reducing recidivism [11]. Building on these insights, the first multisite randomised control trial conducted in the National Probation Service in England and Wales—Mentalization for Offending Adult Males (MOAM) [12]—ensured PPI was central to its methodology. This RCT evaluated the effectiveness of mentalisation‐based treatment in reducing aggression among male offenders with Antisocial Personality Disorder.

The study team collaborated with User Voice, a service user‐led organisation, to involve peer researchers (PRs) with lived experience of serving community or custodial sentences as adults. In the MOAM trial, PRs worked alongside traditional research assistants (RAs) to collect data. The study protocol adhered to best practices for recruitment and engagement, emphasising culturally competent design, trauma‐informed training, and collaborative relationships [13, 14, 15]. Safeguarding procedures and confidentiality protections were implemented [13, 16], drawing on prior research conducted in the criminal justice system with lived experience researchers [17, 18].

The study team hypothesised that involving PRs would reduce barriers to recruitment and retention among participants currently serving a prison or community sentence by fostering common ground between PRs and a population often described as “hidden” in healthcare research [19]. Additionally, this collaboration was expected to facilitate knowledge exchange between PRs and the wider research and clinical team. However, the actual value of including PRs in the trial and the degree to which stakeholders in the RCT agreed on the impact of this PPI approach remains unknown.

This qualitative study aimed to address the following research questions:

1. Explore the MOAM participants' experiences of being interviewed and followed up by a researcher with lived experience of the criminal justice system.
2. Examine the impact of the peer researcher approach on the peer researchers themselves.
3. Investigate the impact of the peer researcher approach on the research and criminal justice professionals involved in the randomised control trial.

## 2 Methods

### 2.1 Design

A semi‐structured interview approach was selected to ensure a comparable data set while allowing participants to freely express their experiences. Each interviewee was asked standardised questions regarding their interactions with PRs and the impact of this approach on the RCT [20]. All participants were provided with a participant information sheet and gave written informed consent before participating in the study.

### 2.2 Participants

Five groups directly involved in the RCT were eligible for the qualitative study:

1. **Trial Participants**: Individuals who consented to participate in MOAM and interacted with either a PR or RA were interviewed to share their experiences of completing self‐report outcome measures with both types of researchers.
2. **Research Assistants (RAs)**: RAs who collected data for the clinical trial but had no lived experience of the criminal justice system were invited to share their experiences of working in a research team that included PRs.
3. **Specialist Offender Managers (SOMs)**: SOMs, responsible for screening and recruiting participants for the RCT and supporting PRs in arranging follow‐up appointments, were interviewed to provide insights from the perspective of criminal justice professionals.
4. **User Voice Operational Staff**: Staff members who line‐managed PRs during the recruitment and data collection phases of the RCT participated to provide a managerial perspective.
5. **Peer Researchers (PRs)**: PRs who collected data for MOAM were eligible to share their experiences and reflections.

### 2.3 Sample

A purposeful sampling approach was adopted [21]. The analysis by Guest et al. informed the number of interviews required to sufficiently understand the experiences of different participant groups. Data collection and analysis continued iteratively until sufficient information power was achieved to address the research questions [22].

### 2.4 Recruitment and Data Collection

Interviews were conducted between April 2019 and March 2020 during the final phase of the RCT by two interviewers without lived experience of the criminal justice system. Participants were initially approached via text message or email. At least 24 h after the initial contact, participants were invited via telephone to take part in the interview, and a paper copy of the participant information sheet and consent form was mailed to them.

Once the signed consent form was returned, the interview was conducted either by phone or in person. Interviews varied in length depending on the participant group, with regular breaks offered as needed. The longest interviews were conducted with PRs and lasted up to 180 min. Interviews with key stakeholders lasted between 60 and 90 min, while interviews with MOAM participants ranged from 10 to 60 min. All interviews were conducted in one sitting, except for one PR interview, which was conducted over two sessions at the participant's request.

The variation in interview length reflected individual preferences. Trial participants were asked about their experiences of interacting with a PR or RA, while key stakeholder interviews focused on experiences of working alongside PRs. An abridged topic guide is included in Table 1 and a copy of the interview schedules in the Supporting Information. Before concluding, participants were invited to provide additional feedback not covered by the interview schedule.

**Table 1:** Topic guide summary.

	Motivation	Defining terms	Training and support	Involvement	Data Collection	Engagement	Boundaries	Collaborative working	Personal skill development	Empowerment
MOAM participants/PR					X	X	X		X	X
MOAM participants/RA					X	X	X		X	X
Peer researchers	X	X	X	X	X	X	X	X	X	X
User Voice operational staff members	X	X	X	X	X	X	X	X	X	X
Research assistants	X	X	X	X	X	X	X	X	X	X
Specialist offender managers	X	X	X	X	X	X	X	X	X	X

All participants received a £35 voucher as a token of appreciation for their participation.

### 2.5 Data Analysis

Interviews were transcribed and analysed by a researcher with no lived experience of the criminal justice system. Thematic analysis [23] was employed to identify patterns of meaning within participant groups and across the entire data set. Initial inductive coding, driven by the data itself, was completed manually, with codes then organised into potential themes. All themes were manually cross‐checked against the extracted codes. The data set was subsequently entered into NVivo 12 qualitative data analysis software, where specific themes were refined to develop an overall narrative. Each theme was defined and named.

### 2.6 Credibility and Validity Checks

To ensure consistency, all data collectors received standardised training before meeting participants. Interview schedules were developed in collaboration with the service user organisation and informed by systematic literature reviews [7, 24]. These schedules were piloted with a nonclinical population with no observable or diagnosable mental health conditions.

To mitigate reporting bias, data collection was conducted by two interviewers not involved in the MOAM trial. Participants were reminded at the start of the interview that their involvement would remain confidential. No participants withdrew from the study, and all were given the opportunity to review their interview transcript before data analysis. Emerging qualitative themes were reviewed by the study team until consensus was reached, ensuring credibility.

## 3 Findings

### 3.1 Demographics

Forty‐seven semi‐structured interviews were conducted across five participant groups:

- **MOAM Participants**: 15 participants who interacted with a PR and 15 who interacted with an RA during the trial.
- **PRs**: All five PRs who collected data for MOAM participated.
- **RAs**: All five RAs involved in MOAM participated.
- **SOMs**: Of the 13 SOMs involved in recruitment, five participated.
- **User Voice Operational Staff**: Two staff members involved in day‐to‐day trial operations participated.

Participant demographics are summarised in Tables 2 and 3.

**Table 2:** MOAM participant demographics.

Characteristics		Contact with PR (*n* = 32)	Contact with RA (*n* = 44)
Age (years)		36.6 (8.6)	36.4 (10.0)
Gender	Male	32 (100%)	44 (100%)
	Female	0 (0%)	0 (0%)
Ethnicity	White British/ White Irish/ White other	25 (78%)	33 (75%)
	Black/ Black British	3 (9%)	6 (14%)
	Asian/ Asian British	1 (3%)	0 (0%)
	White and Black Caribbean/ White and Black African/ White and Asian/ Mixed other	3 (9%)	5 (11%)
Sentence type at baseline	Prison	28 (88%)	42 (95%)
	Community	4 (12%)	2 (5%)
Sentence length at baseline	> 12 months	25 (78%)	31 (70%)
	< 12 months	7 (22%)	13 (30%)

*Note:* Data are *n* (%) or mean (SD).

Abbreviations: PR = peer researcher, RA = research assistant.

**Table 3:** Key stakeholders demographics.

Group		RA (*n* = 5)	SOM (*n* = 5)	UV operational staff member (*n* = 2)	PR (*n* = 5)
Gender	Male	2 (40%)	1 (20%)	1 (50%)	4 (80%)
	Female	3 (60%)	4 (80%)	1 (50%)	1 (20%)

*Note:* Data are *n* (%).

Abbreviations: RA = research assistant,; SOM = specialist offender manager, UV = User Voice, PR = peer researcher.

### 3.2 Themes

The analysis generated 15 themes and 44 sub‐themes, summarised in Table 4 by participant group.

**Table 4:** Themes and subthemes summary.

Participant group	Theme	Subtheme
MOAM participants who met with a peer researcher Total number of interviews completed, *n* = 15	1.1 Trial participants felt understood by the peer researcher	They have been where I am
		It's having a connection with somebody who knows how you feel
		Well they explained stuff to me really well
		Everybody's crimes were different
	1.2 The peer researchers enabled some participants to feel more relaxed	I felt relaxed knowing they'd been in my shoes
		I felt I could be myself
		I just didn't trust the process because of my experience
	1.3 The peer researchers were seen as role models	I held then in high esteem
		I reflected on my own behaviour
		It's given me hope for the future
MOAM participants who met with a research assistant Total number of interviews completed, *n* = 15	2.1 Experience of meeting with a research assistant	Meeting for the first time
		In the room
		Impact of the meeting
	2.2 Reflections on the peer researcher approach	They understand because they have been there
		It depends on the individual person
User Voice peer researchers and operational staff members Total number of interviews completed, *n* = 7	3.1 Skill set	Having that shared experience
		Skills required to be an effective peer researcher
	3.2 Collecting data is prisons and probation offices	Getting in
		Levels of engagement
		Managing boundaries
		Being treated like an ex‐offender
	3.3 Impact on the participant of meeting with a peer researcher	Levels of honesty
		The peer researchers as role models
	3.4 Impact of the role on the peer researcher	Feeling valued and a time to reflect
		Skill development
		Not feeling part of the entire process
		Having that lived experience meant it was more challenging
Research assistants Total number of interviews completed, *n* = 5	4.1 Levels of engagement	I think it's more authentic
		Developing a relationship over time
		It's going to vary
	4.2 Shared learning	Learning experience for the peer researchers
		We learnt so much
		Working together
	4.3 Support and supervision	It was too close to home for some of the peer researchers
		Clinical supervision
Specialist offender managers Total number of interviews completed, *n* = 5	5.1 Breaking down barriers to engagement	It feels like us and them if not
		Any study is just so enriched by the involving service users
		Being empathetic not sympathetic
	5.2 Challenging the ex‐offender stereotype	You just couldn't tell
		Trusted professionals
		Proving change is possible
	5.3 Therapeutic effect of the role of the peer researcher	You haven't been forgotten you are not lost
		He's not an ex‐offender when he comes in
		It can take you back to where you don't want to be

#### 3.2.1 Participant Group 1: MOAM Participants—Peer Researchers

Three themes emerged from interviews with the 15 MOAM participants who interacted with a PR.

##### 3.2.1.1 Theme 1.1: Trial Participants Felt Understood by the Peer Researcher

Participants noted that PRs' shared lived experience fostered understanding and connection. One participant remarked, “Understand your answers a little bit more than someone who's never been in that situation” (MOAM/PR 3). Effective communication skills were highlighted as crucial: “If I didn't understand it, you know, they could recognise that pretty quickly and explained it a little bit more” (MOAM/PR 4). However, some participants expressed concerns about relating to PRs with different offending histories. “We don't really like people like that. Maybe that would be a disadvantage if the person was like a sex offender” (MOAM/PR 14).

##### 3.2.1.2 Theme 1.2: The Peer Researchers Enabled Some Participants to Feel More Relaxed

PRs often helped participants feel at ease, breaking down barriers to engagement. “It made me feel a bit more relaxed, knowing that they had been in like my shoes” (MOAM/PR 1). Conversely, participants expressed hesitation about engaging with PRs who appeared not to have moved on from criminality or who seemed closely tied to the criminal justice system. “Even if they have been in prison, they can still, you know, change and want to be part of that system” (MOAM/PR 9).

##### 3.2.1.3 Theme 1.3: The Peer Researchers Were Seen as Role Models

Participants viewed PRs as inspiring role models given a second chance. “It's just to look at it [the peer researcher], you think fair play” (MOAM/PR 2). Some participants reflected on their own lives as a result of the research process. “It has made me look at life… where I was going wrong and that, the need for direction” (MOAM/PR 1). PRs demonstrated that change was possible, instilling hope and a sense of purpose: “It gives me hope that I can definitely get somewhere myself” (MOAM/PR 4). However, not all participants reported a personal impact. “I think it is good work, and it will benefit a lot of people, even if I don't personally think it will benefit me” (MOAM/PR 14).

#### 3.2.2 Participant Group 2: MOAM Participants— Research Assistants

Two themes emerged from the interviews with the 15 MOAM participants who met with an RA.

##### 3.2.2.1 Theme 2.1: Experience of Meeting With a Research Assistant

Participants initially reported difficulty in trusting the RA due to uncertainty about the process. “At first, I was a bit nervous about what questions they were going to ask” (MOAM/RA16). However, most participants felt more comfortable after the meeting, describing RAs as patient and clear communicators. “They used to speak back to me normal…I didn't feel any pressure” (MOAM/RA30). For some, the process was reflective and even therapeutic. “I find it like therapeutic, like counselling” (MOAM/RA16).

##### 3.2.2.2 Theme 2.2: Reflections of the Peer Researcher Approach

Participants reflected that PRs might better relate to them due to shared experiences. “Until you actually walked in someone's shoes, it's like well you don't really know” (MOAM/RA17). They emphasised the importance of lived experience for researchers collecting data from individuals in the criminal justice system. “If they're a trainee who hasn't had any life experience really…It's like ‘well, what the fuck do you know really?’” (MOAM/RA17).

These reflections echoed findings from participants who had met with PRs, underscoring the potential advantages of lived experience in building rapport. Participants highlighted key skills required for PRs, including strong listening abilities and self‐control. “When someone's got a big ego, they don't like to listen to other people” (MOAM/RA29).

Perspectives on the preferred type of researcher varied:

Some participants valued PRs for their lived experience, which they felt fostered better understanding. “They'd just understand more” (MOAM/RA20).

Others preferred RAs, noting the opportunity for knowledge transfer and the benefit of keeping prison experiences separate from community life. “They're telling me things I don't know, and I can tell them things they don't know” (MOAM/RA28).

A third group expressed no preference, believing the outcome would be the same regardless of researcher type. “It is same end result…I wouldn't feel any different” (MOAM/RA27).

#### 3.2.3 Participant Group 3: User Voice Peer Researchers and Operational Staff

Four themes emerged from the interviews with the five PRs and two User Voice operational staff members.

##### 3.2.3.1 Theme 3:1: Skills Set

Lived experience was identified as essential for understanding participants' perspectives, but it needed to be sufficiently distant to allow PRs to maintain professional boundaries. “You have to be able to take a step back from it…if it's too close, it's too sensitive” (UVPR4). Beyond lived experience, PRs required additional skills to engage participants and collect data effectively. “The idea that anyone with the experience can therefore play a role. Absolutely wrong. People need the skills to do that” (UVOS2).

Effective communication, active listening, and genuine interest in others were critical. “Being able to build a genuine rapport with someone, but communicate on different levels” (UVPR3). Strong leadership skills and the ability to maintain boundaries were also essential for building rapport. “Being able to use your own lived experience to be able to talk about that in an appropriate way, but in a way that inspires and motivates other people to open up” (UVOS2). These findings aligned with trial participants' feedback about the importance of communication skills.

##### 3.2.3.2 Theme 3.2: Collecting Data in Prisons and Probation Offices

PRs faced challenges accessing prisons and probation offices due to staff suspicion of their motives. “Despite the fact that you know obviously MoJ are supposed to have the overall say. It was very difficult” (UVPR4). However, building relationships with local staff helped overcome access barriers. “I had a really good relationship with one prison where I could literally ring them up and go” (UVPR4).

Most participants engaged well, though some felt uneasy meeting with a PR in a probation office, projecting their caution about the environment onto the PR. “They just thought you're another probation officer or another sort of somebody else there to judge them” (UVPR1). Maintaining boundaries was highlighted as essential to mitigate risks for participants, PRs, or other staff. PRs were seen as more vulnerable due to their lived experience. “If you've got someone sitting in front of you that's gone through that, and you've got a similar experience, it's quite heavy” (UVPR5).

PRs were generally treated as professionals, facing similar access challenges as RAs, though some staff exhibited negative attitudes. “You're an ex‐con, you shouldn't be back in here” (UVOS2).

##### 3.2.3.3 Theme 3.3: Impact on the Participant of Meeting With a Peer Researcher

PRs felt they could break down engagement barriers by creating a safe space through shared experiences. “You've got an edge that somebody that hasn't had that lived experience can't have” (UVPR4). These findings supported trial participants' reports of feeling more relaxed with PRs.

Nevertheless, barriers related to trust and fear of judgement persisted for some participants. User Voice noted that many MOAM participants struggled with trust. “If they think you're one of them, because, then they might not want to be honest and admit to things that they think you might judge them for” (UVPR1).

PRs were often seen as role models who had broken the cycle of reoffending. “It's quite amazing to meet somebody that has actually got off, has managed to stop that cycle” (UVPR4). However, this could also lead to demoralisation for some participants who had not achieved similar success. “Why haven't I been able to achieve that?” (UVOS2).

Not all participants felt PRs' lived experience influenced their interaction, with one PR stating, “The role was data collection; it wasn't much more than that” (UVPR2).

##### 3.2.3.4 Theme 3.4: Impact of the Role on the Peer Researcher

PRs reported that the role had a positive impact on their confidence and personal growth. “I was able to do that…having the confidence to go into a room full of people and present to them as an ex‐offender” (UVPR3). The experience allowed PRs to reflect on their own progress. “It made me think about what made me vulnerable and put me at risk of the criminal justice system” (UVPR2).

Collaboration within multi‐agency teams provided opportunities for knowledge exchange between PRs and RAs. “I always felt welcome, well respected…I might learn something from [the research assistant] and then vice versa” (UVPR3).

Despite positive feedback, PRs highlighted challenges, including feeling excluded from certain aspects of the study. “A large part of being a peer was about giving someone a voice, but there was nowhere for our [the peer researchers'] voices to be heard” (UVPR2).

Emotional challenges arose from meeting participants in prisons or probation offices, collecting data, and maintaining boundaries. “It can be quite stressful…particularly if the person you're interviewing has got life experience that's quite negative and it's very similar to your own” (UVPR4). User Voice noted that PRs had developed coping mechanisms to manage these challenges. “[The peer researchers] have kind of this capacity to cope with that somehow” (UVOS1).

#### 3.2.4 Participant Group 4: Research Assistants

Three themes emerged from the interviews with the five RAs who worked alongside the PRs.

##### 3.2.4.1 Theme 4.1: Levels of Engagement

RAs observed that the involvement of PRs positively impacted engagement levels and facilitated more authentic interactions with participants. “[The peer researchers] get more; get good quality data from participants in terms of the honesty at times” (RA2). The importance of consistency and confidentiality was highlighted to ensure participants understood that PRs were not part of the criminal justice system. “It's very important that the peer researchers are able to make it clear that this is all confidential—we're not going to hand anything over” (RA1).

These findings aligned with reports from trial participants who met with PRs and emphasised the value of clear boundaries. However, RAs noted that engagement levels were not universally improved, as some participants were indifferent to shared lived experience. “I think they either trust you to not hand over [the information] or they don't” (RA4).

##### 3.2.4.2 Theme 4.2: Shared Learning

RAs noted that PRs developed confidence and skills in research, organisation, and communication through their role. “Recognising the unique kind of extra skill, they have from their own lived experience” (RA1). These observations echoed the PRs' own accounts of increased confidence.

Working alongside PRs was a positive experience for RAs, offering opportunities for mutual knowledge exchange. RAs improved their research and engagement skills and gained a deeper understanding of the criminal justice system. PRs' local knowledge was particularly valuable. “They taught us just how much the prisons have changed as well over the years and how much more understaffed they are” (RA5).

##### 3.2.4.3 Theme 4.3: Support and Supervision

RAs highlighted that meeting participants in a probation office or prison could be triggering for PRs, especially when visiting a prison where they had previously served time. The emotional impact varied depending on how much PRs had moved on with their lives. “You know if it brings up stuff for them that they've not had a chance to think about in a while, or it's still quite painful for them, I think it could be quite difficult” (RA1).

These findings supported User Voice's assertion that lived experience should not be too recent and that maintaining boundaries was critical for PRs. RAs felt that PRs required greater emotional support in their roles, describing the existing supervision as insufficient. “They [the peer researchers] might need more supervision and support, or space for that because of the parallels there might be” (RA1).

#### 3.2.5 Participant Group 5: Specialist Offender Managers

Three themes emerged from interviews with the five SOMs who were part of the clinical team and worked alongside the PRs.

##### 3.2.5.1 Theme 5.1: Breaking Down Barriers to Engagement

SOMs described how PRs were effective in breaking down barriers to engagement with participants. “It became really hard to maintain that contact with him. It was almost when he spoke to the peer researcher, it reminded him that he wanted to talk to us [Mentalization‐Based Treatment team] as well” (SOM4). PR involvement facilitated knowledge transfer between PRs and SOMs. “What we've got to do is learn and think about, in terms of a service user's experience, what might help them” (SOM1).

However, the SOMs emphasised the importance of PRs maintaining a clear boundary between their former and current lives to safeguard against being drawn back into criminal activity. “You know it's a powerful life… so I do think they really need that degree of separation for their own safety” (SOM4). These findings align with reports from RAs and User Voice interviews, which highlighted the significance of boundaries and supervision to mitigate the emotional impacts of the role.

##### 3.2.5.2 Theme 5.2: Challenging the Ex‐Offender Stereotype

The SOMs noted that, in most research sites, PRs were trusted and treated as professionals. However, they also recounted instances where PRs faced suspicion and unequal treatment. “I remember they weren't allowed in the office, and some of the service users are given a little talk at the team meeting” (SOM1). These observations mirrored the access challenges described by PRs in their own interviews.

Despite these challenges, PRs were seen as proof that meaningful change was possible for MOAM participants and the wider probation service. “That person being allowed to come in must mean they've really made it, because they really have. They've crossed that divide” (SOM1).

##### 3.2.5.3 Theme 5.3: The Therapeutic Effect of the Role for the Peer Researchers

The SOMs highlighted the dual benefit of the PR role: participants felt valued and hopeful after interacting with PRs, while PRs themselves experienced personal growth and validation. “They found it empowering that someone was in that position interviewing him” (SOM1). PRs described feeling rewarded and respected through their contributions. “Big boost to self‐respect and you know, how they see themselves. They are not an ex‐offender when they come in” (SOM4).

These findings align with trial participants' reports of PRs as role models. However, SOMs also raised concerns about potential emotional challenges for PRs, particularly when meeting participants in custody. “You hear information that takes you back to where you were yourself in prison, and sometimes that's tough” (SOM2). These concerns underscored the importance of providing PRs with adequate supervision to support their emotional well‐being.

## 4 Discussion

Despite the limited evidence on the effect of PPI in clinical trials within forensic settings, MOAM represents the first attempt to explore the impact of involving researchers with lived experience as data collectors in a multisite RCT conducted within the National Probation Service in England and Wales. This approach aimed to reduce barriers to participant recruitment and retention by fostering common ground with a population that often struggles to engage in research [7] while facilitating knowledge transfer between key stakeholders.

Overall, MOAM participants reported feeling at ease with PRs, who created a safe space that potentially strengthened the breadth and depth of the data collected. Stakeholder interviews supported these findings, noting that the involvement of researchers with lived experience generally helped participants feel more relaxed. However, PR involvement did not always guarantee engagement. Some participants expressed concerns about PRs, fearing they might still be involved in criminal activity and thus could not be trusted. These findings align with Livingston et al.'s study of treatment planning in a forensic mental health hospital, which found that some participants were reluctant to disclose information to researchers with similar lived experiences [25].

In contrast, stakeholder interviews did not raise concerns about trust but emphasised the importance of ensuring adequate separation between PRs' former and current lives for their own safety and well‐being. Some participants cited pervasive mistrust stemming from their own lived experiences as a barrier to engagement. This pattern of mistrust aligns with personality traits associated with Antisocial Personality Disorder [26] and the effects of imprisonment [27], which can generalise across relationships and interactions. These factors should be considered when interpreting the study's findings.

All interviewees highlighted external factors—such as the environment, the nature of lived experience, and the attitudes of some staff members—as important considerations when evaluating the impact of PRs. Communication and organisational skills were universally emphasised as essential for researchers, regardless of their level of lived experience. Despite these challenges, most PRs found their role meaningful and believed they contributed valuably to the trial's implementation.

The qualitative accounts revealed that the inclusion of PRs created opportunities for knowledge transfer, challenging stereotypes associated with being an “ex‐offender” and positioning PRs as role models. By drawing on PRs' experiential knowledge, the study team helped break down barriers created by the “us and them” culture. This suggests that knowledge gained through lived experience can enhance clinical trials, offering insights not replicable through academic expertise alone.

RAs reported improvements in their research and engagement skills and a deeper understanding of the criminal justice system through collaboration with PRs. SOMs described how PRs enriched their understanding of the service user experience, while PRs benefited from learning through their participation in a multi‐agency team. Standpoint theory provides a framework to understand this dynamic, suggesting that the knowledge of traditionally excluded individuals is validated through PPI, as peers contribute unique perspectives inaccessible to traditional researchers [28].

PRs felt empowered by their roles, as their knowledge was valued in discussions with academic staff, supporting Arnstein and Boote's models, which argue that higher levels of participation lead to greater redistribution of power [29, 30]. SOMs suggested that future studies should more fully embed PRs in the research design to maximise their impact. These findings align with Rise et al., who demonstrated that user participation strengthens the authenticity of research outcomes [31], and support prior evidence on the value of PPI in RCTs [32, 33, 34, 35].

The MOAM case study demonstrates that involving researchers with lived experience in clinical trials can bridge the gap between service users and professionals by creating common ground and facilitating knowledge transfer. This approach can also address broader structural power imbalances, benefiting individuals, science, and society.

## 5 Strengths and Limitations

This study is the first to explore the impact of PPI on an RCT within a forensic setting in England and Wales. The involvement of lived experience guided the study's aims and design, highlighting its innovative approach. However, the findings must be interpreted in light of several limitations.

The study focused on a highly stigmatised population, and the findings may not generalise to other contexts. The study involved one service user organisation and five PRs, meaning the reported PPI impact could be specific to these individuals and challenging to replicate in different settings. Data were collected at a single time point during the final phase of the RCT, potentially limiting the results due to recall bias.

Moreover, the MOAM participants interviewed had not exclusively interacted with either PRs or RAs during the follow‐up period, which may have influenced their descriptions based on prior encounters with different research team members. Data analysis was conducted by a single researcher, which may have constrained the interpretation of the findings.

Interviews were conducted by RAs with no lived experience of the criminal justice system, which could have introduced further barriers to engagement. Future research should evaluate the impact of PPI across multiple case studies within a single evaluation to enhance generalisability and work collaboratively with lived experience colleagues to optimise the benefits derived from their involvement.

## 6 Conclusion

This study explored the impact of involving PRs in the first RCT conducted within the National Probation Service in England and Wales, capturing perspectives from trial participants, RAs, SOMs, and the PRs themselves. Researchers with lived experience helped to break down power differentials and engage participants who often struggle to take part in research. The methodology bridged the gap between two communities, creating opportunities for knowledge transfer while challenging conventional ideas about knowledge valuation and stereotypes associated with being an “ex‐offender.”

However, PPI did not universally break down barriers to engagement. For some participants, it introduced new challenges, as trust issues arose when PRs were perceived as part of the authoritarian system.

The findings underscore the importance of soft skills, particularly clear communication, and highlight the need for adequate clinical supervision to support PRs in managing the emotional demands of their role. More broadly, the study challenges assumptions about knowledge definition and who is best suited to study whom. These findings provide a foundation for best practice in implementing PPI across diverse research contexts, regardless of the type of lived experience involved.

## Author Contributions

**Elizabeth Simes:** conceptualisation, investigation, funding acquisition, methodology, writing–original draft, formal analysis, data curation. **Stephen Butler:** writing–review and editing, supervision, conceptualisation, methodology, funding acquisition, investigation. **Elizabeth Allison:** writing–review and editing. **Barbara Barrett:** writing–review and editing, funding acquisition. **Anthony Bateman:** writing–review and editing, funding acquisition. **Angus Cameron:** writing–review and editing, funding acquisition. **Mike Crawford:** writing–review and editing, funding acquisition. **Alison Frater:** writing–review and editing, funding acquisition. **Zoe Hoare:** writing–review and editing, funding acquisition. **Mary McMurran:** writing–review and editing, funding acquisition. **Paul Moran:** writing–review and editing, funding acquisition. **Stephen Pilling:** writing–review and editing, funding acquisition. **James Wason:** writing–review and editing, funding acquisition. **Jessica Yakeley:** writing–review and editing, funding acquisition. **Peter Fonagy:** supervision, writing–review and editing, conceptualisation, methodology, funding acquisition, investigation.

## Ethics Statement

The study protocol was approved by the London – South East Research Ethics Committee (14/LO/1696) and the National Offender Management Service (2014‐315). The trial sponsor, University College London, played no part in the study design; collection, management, analysis, and interpretation of data; writing of the report; or the decision to submit the report for publication.

## Conflicts of Interest

The authors declare no conflicts of interest.

## Supporting information

Supporting Information.

## Data Availability

The data that support the findings of this study are available on request from the corresponding author. The data are not publicly available due to privacy or ethical restrictions.
