# Supplementary material for: Bridging the Gap: A Qualitative Study Exploring the Impact of the Involvement of Researchers With Lived Experience on a Multisite Randomised Control Trial in the National Probation Service in England and Wales
Source: Health Expect. 2025 Feb 4;28(1):e70162. doi: 10.1111/hex.70162 (PMC11791406; doi:10.1111/hex.70162)
Supplement: Supplementary file 1 — Supporting Information. [file HEX-28-e70162-s001.docx]

**Interview schedule: MOAM participant**

Thank you for agreeing to take part in the interview. This interview is about your experience of meeting with a researcher to complete the questionnaires. The interview will take approximately 1 hour and you will be free to take a break at any time. You do not have to answer every question during the interview, and you only have to share the information you would like to share.

Do you have any questions before I turn on the Dictaphone to record the interview?

[answer any questions raised]

I am now going to turn on the Dictaphone to record the interview

[Turn on the Dictaphone]

[State date of the interview and participant ID number]

We are now going to start the interview

**Engagement / Data collection:**

The first couple of questions are about meeting with the researcher.

**Q1. How was your appointment arranged?**

PROMPT: How did the researcher contact you?

PROMPT: Was your offender manager involved?

PROMPT: Who decided what time and when you were going to meet?

**Q2. How would you describe the experience of meeting with the researcher to complete the questionnaires?**

PROMPT: [Only to be asked to the participants who met with a peer researcher] Did the peer researcher tell you they had personal experience of being in prison or on probation, and how did you feel about this?

PROMPT: Did you feel listened too?

PROMPT: Did you feel understood?

PROMPT: Did the researcher make you feel comfortable?

PROMPT: What was it like meeting with a researcher in a probation office/prison to complete the questionnaires?

PROMPT: Was there anything about the interaction that made you feel uncomfortable or you didn’t like?

**Q3. How did you feel about answering the questions?**

PROMPT: Did you feel you were able to be honest?

PROMPT: Did you feel you were able to trust the researcher?

**Q4. Did you answer every question or were there questions you didn’t want to answer?**

PROMPT: How did you feel about this?

PROMPT: How did the researcher respond?

**Q5. How would you feel about meeting the researcher again in the future to complete the research questionnaires?**

**Boundaries:**

Some of the researchers collecting data for the project have lived experience and have either been in prison, on probation or both. These researchers are called peer researchers.

Some of the researchers don’t have lived experience and are called research assistants.

**Q6. What do you think are the advantages of someone with lived experience meeting with participants to complete the questionnaires?**

PROMPT: Would you prefer to meet with a researcher who did have a shared experience? If so why?

PROMPT: Would you feel more comfortable?

PROMPT: Does it make a difference?

**Q7. What do you think are the disadvantages of someone with lived experience meeting with participants to complete questionnaires?**

PROMPT: Would you prefer to meet with a researcher who didn’t have a shared experience? If so why?

PROMPT: Would you feel more comfortable?

PROMPT: Does it make a difference?

**Empowerment/personal skills and development:**

**Q8. In what ways do you think meeting with the researcher to complete the questionnaires has had a positive impact on you?**

PROMPT: Has the experience effected the way you see the future?

PROMPT: Following this experience, do you think you would like to be part of another research project in the future?

**Q9. Can you tell me a bit about the more difficult aspects of meeting with a researcher to complete the questionnaires?**

PROMPT: Is there anything you didn’t enjoy?

**Q10. Before we bring interview to an end, is there any other feedback you would like to share with me about your experiences of meeting with the researcher to complete the questionnaires?**

Thank you for participating in the interview. We have now reached the end of the interview and I am now going to turn off the Dictaphone.

[Turn off Dictaphone]

**Interview schedule: Key stakeholders**

Thank you for agreeing to take part in this interview. As outlined in the participant information sheet this interview is about your experiences of working alongside the User Voice peer researchers on the MOAM trial. The interview will take up to approximately 1 hour and you will be free to take a break at any time during the session. You will be asked questions about your experiences of working on the MOAM trial and the impact of the peer researcher approach on the trial. You do not have to answer every question the researcher asks you during the interview, and you only have to share the information you would like to share.

Do you have any questions before I turn on the Dictaphone to record the interview?

[Answer any questions raised]

I am now going to turn on the Dictaphone to record the interview

[Turn on the Dictaphone]

We are now going to start the interview.

**Motivation**

**Q1. Can you tell me about why you think the peer researcher approach was adopted for the trial?**

**Q2. Why do you think someone with lived experience of the criminal justice system might be motivated to apply for the role of a peer researcher?**

**Defining terms:**

**Q3. What is a peer researcher to you?**

PROMPT: Who can be a peer researcher?

PROMPT: What skills do you think you need?

**Q4. How would you describe the peer researchers’ job in the trial?**

**Training and support**

We are now going to move on to talk about the training and support offered to the peer researchers.

**Q5. From your observations what type of support and training do you think the peer researchers need in their role?**

**Q6. Can you tell me about any additional training or support you think the peer researchers would benefit from for this project, and why?**

**Involvement:**

**Q7. How would you describe the peer researchers’ role in the trial?**

**Q8. Are there areas of the trial the peer researchers have not been involved in, and how do you feel about this?**

**Data collection:**

Part of the peer researchers’ role involves meeting with participants to collect follow up data.

**Q9. Can you tell me about how you think the peer researchers experience collecting data in a probation office?**

**Q10. How do you think the participant experiences meeting with the peer researcher in this type of space?**

**Q11. And how do you think the peer researchers’ experiences collecting data in a prison?**

**Q12. How do you think the participant experiences meeting with the peer researcher in this type of space?**

**Engagement:**

For the trial the peer researchers are meeting with participants in different parts of the country. **Q13. From your experience of working alongside the peer researchers/being a peer researcher can you tell me about how they approach engaging participants?**

**Q14. Can you tell me about a time when you think the peer researchers/you might have found it difficult to engage a participant?**

PROMPT: Why do you think the peer researchers might find it difficult to engage with some participants?

**Q15. From your observations how do you feel about the honesty of the information disclosed to the peer researchers by participants?**

**Q16. For those participants who have met with a peer researcher more than once how would you describe their engagement over time?**

**Boundaries**

**Q17. What do you think are the advantages of someone with lived experience collecting data for the trial?**

**Q18. What do you think are the disadvantages of someone with lived experience collecting data for the trial?**

**Collaborative working**

For this project I understand you are working with lots of different groups of people, who work for different types of organisations.

**Q19. What is it like working on a research project with a user led organisation?**

PROMPT: Can you describe a situation when you think it has worked particularly well?

PROMPT: Are there times when it has been more challenging, and can you give me an example?

**Q20. How does it go when the peer researchers need to communicate with clinical staff who are part of the MBT team?**

PROMPT: Can you describe a situation when you think it has worked particularly well?

PROMPT: Are there times when it has been more challenging, and can you give me an example?

**Q21. And how does it work when the peer researchers need to communicate with the offender managers in each of the sites?**

PROMPT: Can you describe a situation when you think it has worked particularly well?

PROMPT: Are there times when it has been more challenging, and can you give me an example?

**Personal skills and development**

**Q22. What new skills do you think the peer researchers have learnt since working on the project that they didn’t have before?**

**Q23. From your experience what influence do you think interactions with the peer researcher has had on the participants? (MBT group members, the men that are involved in the trial)**

PROMPT: Do you think the experience of meeting with a peer researcher might support the participant to rehabilitate?

**Q24. What do you think the peer researchers and User voice have taught UCL about conducting research in the criminal justice system?**

**Empowerment**

**Q25. In what way do you think the trial has had an impact on the how the peer researchers feel about themselves in a positive way?**

**Q26. Can you tell me a bit about the more challenging and difficult aspects of the peer research role?**

**Q27. How would you describe the impact of the peer research approach on the participants?**

**Q28. What have you enjoyed most about the experience of working alongside the peer researchers/working as a peer researcher?**

**Q29. Before we bring the interview to an end, is there any other feedback you would like to share with me about the impact of the peer researcher approach on the trial?**

Thank you for participating in the interview. We have now reached the end of the interview and I am going to turn off the Dictaphone.

[Turn off Dictaphone]
